# Teaching and Learning in COVID-19: Pandemic Quilt Storying

**DOI:** 10.1177/19408447231169069

**Published:** 2023-07-12

**Authors:** Jenny Ritchie, Louise G. Phillips, Cynthia Brock, Geraldine Burke, Melissa Cain, Chris Campbell, Kathryn Coleman, Susan Davis, Esther Joosa

**Affiliations:** 1 Te Puna Akopai School of Education, Te Herenga Waka Victoria University of Wellington, Wellington, New Zealand; 2 Chair of Discipline (Initial Teacher Education), 228556 Southern Cross University, Bilinga, QLD, Australia; 3 Wyoming Excellence in Higher Education Endowed Chair in Literacy Education, 4416 University of Wyoming, Laramie, WY, USA; 4 School of Curriculum, Teaching and Inclusive Education, Faculty of Education, 2541 Monash University, Melbourne, Australia; 5 National School of Education, Faculty of Education and Arts, 95359 Australian Catholic University, Brisbane, QLD, Australia; 6 Division of Learning and Teaching, 1109 Charles Sturt University, Canberra, ACT, Australia; 7 Melbourne Graduate School of Education, University of Melbourne, Australia; 8 School of Education and the Arts, 625191 Central Queensland University, Rockhampton, QLD, Australia; 9 Education Consultant, Singapore

**Keywords:** COVID-19, collaborative methodology, collaborative storying, quilting as methodology

## Abstract

Something changed during the pandemic; we attuned to a call. A call to action, breathing, support, activism, care, well-being, community, minimised mobilities, planetary health and our relations to all these things, and more. We are women working in education spaces across multiple communities, responsive to ongoing matters of concern (Latour, 2008), aware that our rhizomic connections have no middle or end. We use the method and metaphor of the quilt in this collaboration and hold quilting as a Feminist intervention, a return to her-stories and ways of knowing through story as we stitch together cultural and material stories of place. Our COVID-19 chronicles are a creative, collaborative exploration of the initial impact of the COVID-19 pandemic on learning and teaching across our respective countries. This paper is a collaboration of critical auto-ethnographies (Holman Jones, 2016), quilted and stitched together by a group of education scholars who united to research the impact of online emergency teaching that forced education site closures globally. Through this collaborative image quilting, we curated responses to our initial 100-word stories of pandemic life in 2020, that we had posted on a collaborative Padlet. Feminist, storying, and ethnographic theory inform alignment and stitching of each 100-word patch.

## An introduction

Our varied experiences of the pandemic, as academics, parents, friends, and community members, were of course nuanced and complex. Yet our personal quilted COVID-chronicles resonated, generating a sense of connection. This scholarly community might have been unexpected, since we come from a range of countries and communities, yet simultaneously expected, since our circles of scholarship are informed by shared matters of concern (Latour, 2008) in education and educational research. As we collected data, curated, analysed, and storied the data of the international participants in our research project survey (Phillips et al., 2021), we too saw ourselves reflected in the pandemic storying gathered (Holman Jones, 2016).

From October to December 2020, a Padlet portfolio hosted our pandemic storying via hundred-word patches, hereafter called ‘hundreds’ (Anderson et al., 2022; Berlant & Stewart, 2019; Healy & Edwards, 2020). In early 2022, we once again came together prompted by a further call to gather for retrospective reflection. Our methods of gifting and patching have developed within our Collaboratory as we have seen how the data impacts, shifts, turns, and mirrors itself. Seeing the data differently has offered us new ways of thinking with storied data, and the Padlet afforded us a new form as we used the Tufte (2020) image quilt built into the Padlet as an archive method (Healy & Coleman, 2022).

## Rebounding

We resume our collaboration, in the context of both ongoing and new crises. Our stories hold new sorrows and concerns. We resume storying our quilt as the world faces a new war, a new strain and accompanying wave of COVID-19, untenable staffing shortages and related workloads, and educational limits that we all find individually challenging. Witnessing angry protests against health measures we question our effectiveness as educators in the face of the impacts of dis/misinformation, manifesting in community confusion and alt-right fuelled dis/misinformation conspiracies. We seek and hope for compassion, empathy, and honour. We look to what makes us human.

## Quilting the Stories (Methodology)

We use the method and metaphor of Image Quilts from Tufte and Schwartz (http://imagequilts.com/) and hold this term as a Feminist intervention, acknowledging the intercultural legacy of women’s quilt-making (Belton, 2021; Fitz Gerald, 2003; Thomas, 2018). In doing so, we claim a return to (her)stories and ways of knowing through story. Here we quilt another story, drawing from African American cultural critic, bell hooks (2009). Hooks observed her maternal lineage of quilting with concentrated focus on placement and pattern, creating visual histories with curated remnants of family members’ clothing. Each fabric shape holds embodied lived stories, imbued with ‘self-reliance and self-determination … aroused by quilt-making’ (p. 166).


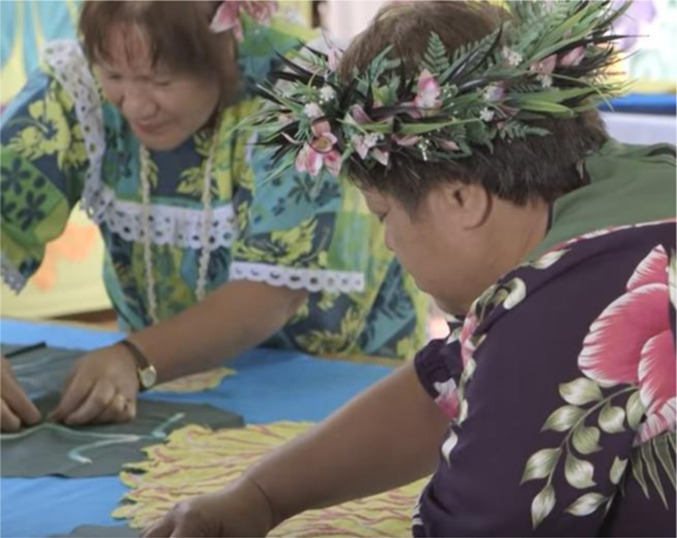


***Tīvaevae: Stitched with*** ***lov*** ***e***

See video at: https://www.youtube.com/watch?v=EjGuCaZatPU&ab_channel=MuseumofNewZealandTePapaTongarewa

Our collaborative, piece-by-piece quilting joins together the spaces between our dis/connections. As with Tivaevae, ceremonial quilts from the Cook Islands, we value place, time, location, and intergenerational sharing. Tivaevae are ‘a visual manifestation of Cook Island beliefs, customs and mana’ (Kea, 2009, p. 29) which story memories of homelands, depicting joyful, colourful plants and flowers. A language of love*,* carefully and creatively crafted by women, Tivaevae are presented as ceremonial gifts of the intergenerational transfer of memory and aroha (love) for extended family and community. They have great poignancy for people from the Cook Islands who now live in Aotearoa.

We utilise the method of quilting to piece and stitch together our pandemic education stories. Our quilt’s initial patches were automatically stitched by an AI Image Quilt drawn from our autoethnographic storying Padlet as we began our wider collaborative project, the *Teaching and Learning in COVID-19 Times Study* (Phillips et al., 2020). This initial quilting has since been undone, rethought, and pieced back together in ways that evoke our collaborative narrative. Our autoethnographies now form a pandemic patchwork quilt, a vibrant and beautiful symbol of the care, compassion, and honouring that served as a stabilising thread throughout our research journey.

**Quilting as method**, or ‘QAM’ as explained by Sonia Arellano (2022), is informed by Feminist-materialist methodologies and by scholarship that reflects a holistic view of the world and its human and more-than-human co-habitants. It entails processes of mapping embodied everyday practices, positioning ‘stories, relationships to the land and local communities, and accountability to people’ (Arellano, 2022, p. 18). Quilt-making traverses cultures and classes, collectively evoking a sense of democratic community, gifting warmth, beauty, well-being, and creativity amongst other things. We know that stories live in quilts, revealing cultures of place and an aesthetics of transformative power (hooks, 2009, p. 168).

During the pandemic, as our group met to discuss and analyse the data from our ‘*Teaching and Learning in COVID-19 Times*' survey, in a parallel process we also collected our own stories in our Padlet. These short stories were drafted as reflections of life as they pushed and pulled at our senses of ourselves. Then slowly, we began to quilt these together as a way of mending our experiences and piecing together the slippage of time. The method of quilting affords time; slowing down time; allowing pause for thought. It is a generative and restorative arts practice, enabling collaborative re-storying.

## Quilting to a Pattern of Hundreds

There are diverse ways to quilt, there are cultural and material stories of place held deeply in this practice. They are gifts that hold a narrative and gather narratives as they traverse spaces. A quilt holds individual pieces that are patched together; stitched and mended, folded and matched. Our QAM is affective autoethnography, stitching together stories written individually into a collaborative quilt. We apply Lauren Berlant and Kathleen Stewart’s (2019) proposition of writing in a hundred words, as the central pattern for quilting. Berlant and Stewart’s collaborative text demonstrates affect theory as practice through 100-word pieces, or multiples of hundreds.

Each patch is constrained to one hundred words as in Berlant and Stewart’s book ‘The Hundreds’ (2009). This protocol tailors our contributions within the whole composition, with attention to meaning-making (MacDonald et al., 2022). Through the crafting, we respond to one another, consider what to preserve, and what is redundant. We adopt a convention of identifying each quilter or story-maker with their name, and sometimes the date, in brackets after each hundred. We do this to show the values and ways of knowing respectively held. Key emboldened words stitch the patches of hundreds together across people, communities, time, and places.

## Herewith, our COVID storying patches…

**A fraught quest** home plotted with numerous false starts, whilst I continue to teach, research, and support students and staff amidst widespread uncertainty. Student study plans are in chaos, and no other section of the university understands the ramifications of school closures and restrictions on nullifying student teacher placement progress. I am traumatised by five flight cancellations across four months to return home to my husband and sons. Impact = two hospitalisations, six months of strong sleeping tablets, and four general anaesthetic surgeries. **Home** is felt through smell, through familiarity, through comfort, through reassurance, through **belonging** and identity, and through security (Louise, October 2020).


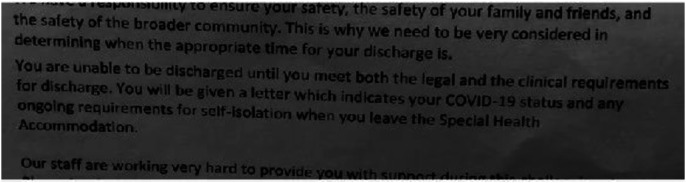


***Quarantine discharge instructions***

The concept of **home** shifted during the initial stages of the pandemic. From home we were now teaching, marking, meeting, designing, and developing curriculum. At home we set up home offices, created new sites for teaching so that we had walls behind us, set up lighting, and bought new ergonomic chairs. Home had been a place of **refuge** away from teaching before the pandemic, it was separated not just physically from where we taught but emotionally. I used to get home and take off my school clothes, now I was teaching with no shoes and the dog at my feet (Kate).

It has now been 18 months since I **returned home** and cocooned myself in familial belonging. I am immersed in the soothing comfort of home and local community with dwindled desire to go beyond my safe swathed microcosm. I successfully secured a permanent position at a local university about a year ago, so I could abandon my overseas appointment and insurmountable frustrations I wrote of at the end of 2020. Now that travel bans have lifted, international research is possible again, and the day after tomorrow, I return for research to the nation I traumatically fled from 18 months ago (Louise, 29 May 2022).

### Changing Times

At my university, we are now undergoing a university-wide restructure with widespread **uncertainty**. Staff who are working at home seem happy they are away from the office. I just assisted a staff member who was worried about the VER (Voluntary Early Retirement) as she doesn’t want to take it but felt she might miss out if she doesn’t. She also doesn’t want to be redeployed, and the amount of **change** she will need to undergo is so unknown. She is happy she is still working at home – even though we don’t really have COVID in the state (Chris, 16 October 2020).

**UNCERTAINTY**… The current topic of conversation at the University of Wyoming is the extent to which the **phased re-opening** at UW will continue. University officials have worked diligently this past summer to develop a plan for the phased **re-opening**. The goal was to keep everyone safe whilst also giving students and professors the opportunity for some face-to-face interaction. Yet, pockets of COVID-19 have broken out. People are being quarantined. The phased **re-opening** was supposed to be continued throughout Thanksgiving celebrations in the US. If we get too many cases, we will go back to all online. Change and uncertainty abound (Cynthia, October 2020).

Evidently, we had the choice to stay on campus or teach from home. I put the vote to my tutorial, and they voted to return. It was week four, and we had begun to form **relationships**. We enjoyed working together and looked forward to more. By the time I reached home, the email arrived announcing that classes were cancelled and only ‘essential workers’ were allowed back on campus. But wait! I had left food in my fridge. I hadn’t watered my beloved plants on the windowsill. I can be flexible but can’t cope with **plans changing** at the last minute (Melissa, March, 2020).

### A Gradual Release

On 7 April 2020, Singapore announced a lockdown and schools moved to home-based learning. Called a ‘circuit breaker’: it felt like an electric **shock**. It bolted me, not knowing how it would impact my life and work in the arts, especially with students with disabilities. Now in October, there is a gradual easing of rules, especially for schools. Being able to come back, I can see the effect of real engagement and observe the value of physical interactions and immediate responses. Despite many sensory challenges, students hold on to their masks. For them it has become part of their uniform (Esther, October 2020).


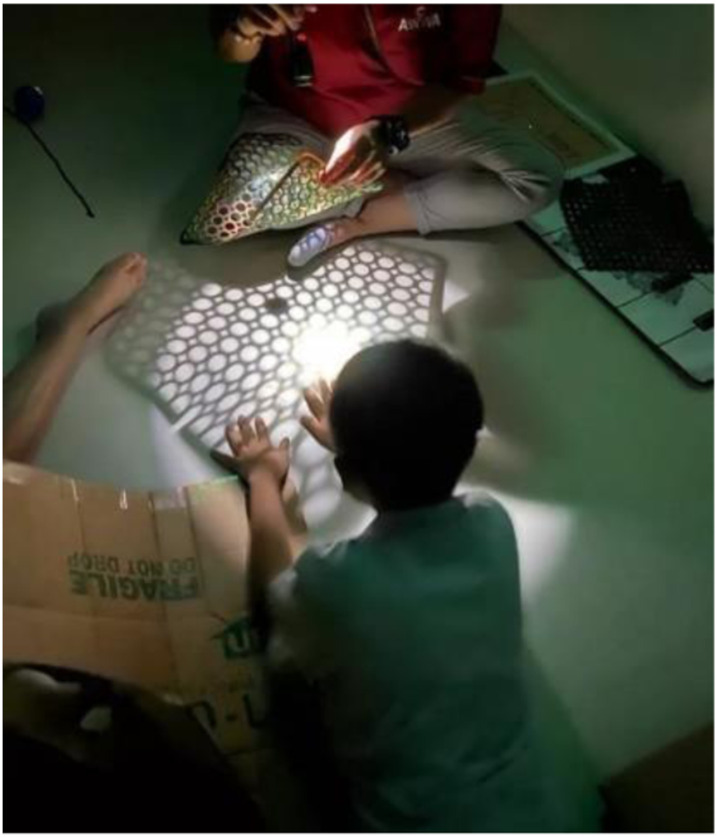


***Young*** ***mask-*** ***wearing*** ***child engaged in light*** ***play. Photo: Esther Joosa***

I begin to get slower and slower. Finding it difficult to balance, I **succumb** to ambulating with a walker. Negotiating something happening to my body while trying to continue the positive start I had to the semester when we were on campus. Sparky as soon as I turn on the camera for my tutorials, but desperately ill when I sign off. Three weeks in hospital and still working from bed. I need an operation, but all elective surgery is postponed indefinitely. Eventually the hospital relents, and I have the cure that saves me. But with only four days off work (Melissa, May, 2022).

I look back at a year that started with carefree travel, a sudden lockdown, and a gradual release. It taught me the value of the **home** and **releasing through creativity**. Nothing can beat creative experiences of touching, feeling, seeing, and responding. With the home as the genesis of everyday learning, such as a plastic bathmat for light play, brings parents into the conversation about home art experiences. Education is not just about the subject we teach or roles we have but to understand that COVID-19 affects social life, relationships, finances, and can have an effect on health, and emotional health (Esther, December 2020).

To patchwork a quilt, we stitch and sew pieces to create a **pattern**, placing creative **connections** between pieces. Here I am stitching between spaces, as the idea of the toss reverberates in my thinking and reminds me of the language of teaching and learning in the pandemic. We pivoted, shifted, jumped, caught, and moved. Terms that have never before really resonated with me, but here I feel their actions, activities, and affects. Their movements and energies remind me that ‘pedagogy enacted with/in a relational ontology embraces what pedagogy can do rather than what it is’ (Healy & Mulcahy, 2021, p. 559).

***MANAGING COVID??*** Are we really managing it??? COVID-19 numbers are on the upswing in Wyoming and across much of the US. A biblical reference in the Old Testament describe**s** the Israelites as **‘**a stiff-necked people**’**, which seems to pertain to so many current US Americans. Whereas some US communities and groups are following important **precautions**, many are not; hence the **predicament** in which we find ourselves. We are approaching one of our most important holidays of the year (Thanksgiving), and many will not be able to celebrate this Thanksgiving with their families and friends because of the rising numbers of COVID-19 cases (Cynthia, November 2020).

### Kindness Courage Optimism

The image below frames our experience of the pandemic in Wyoming. Thousands of these signs were placed in yards and businesses across the state. My university sought to reinforce the message that we could make it through the pandemic. Though many strove to remain **kind, courageous,** and **optimistic** during this difficult time, many did not make it through unscathed. We had our share of ‘fits and starts’ as friends and colleagues lived through isolation, lost their positions due to budget cuts, and some even lost their lives. We carried on, and we are carrying on, even amidst sadness and difficulty (Cynthia, June 2022).


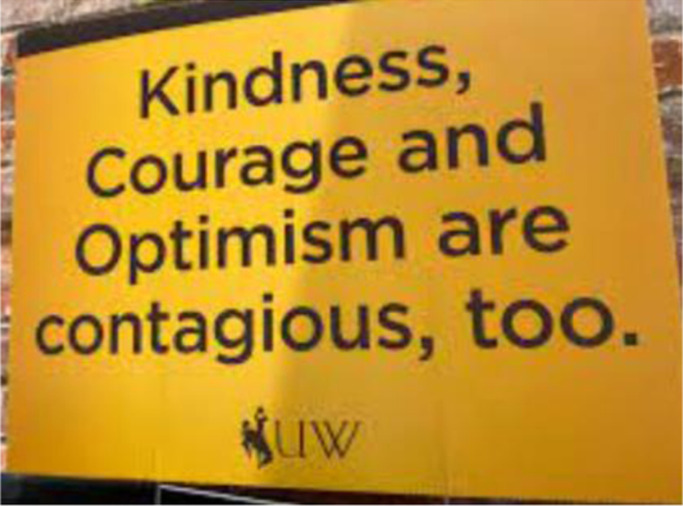


***Kindness, Courage, Optimism, and Contagion***

These words resonated as I recalled how our Prime Minister Jacinda Ardern had been determined to lead a government committed to **kindness** and **empathy**. She retained her determination as she led our nation through the past two and a half years of the pandemic despite the vilification of vocal anti-vaccination protesters. The latter have been infected with a different **contagion**, that of the disinformation of conspiracy theories imported from overseas via social media feeds laced with misogyny. These disinformed protestors occupied our parliament grounds for 23 days this February, some aligning Jacinda Ardern with Hitler (Jenny, Wellington, 19 June 2022).


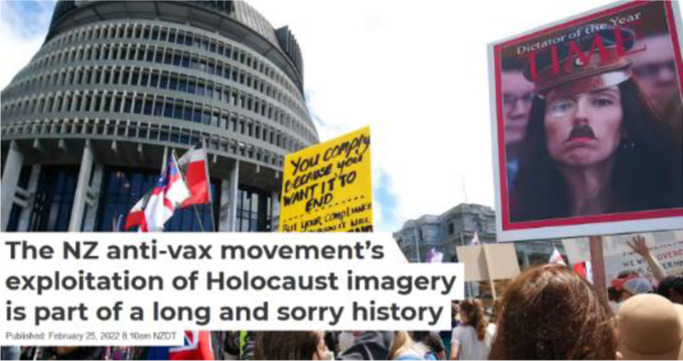


***Anti-vax protesting in Wellington, Aotearoa New Zealand. S**ource:* (Lichtner, 2022)

Semester Two – back on campus. There’s no substitution for learning together in the same physical space. These nuances are hard to express; unspoken attunement in recognition of each other. After spending way too much time on a computer in semester 1, I was excited (overjoyed!) to meet my new classes in semester 2. But once in the room and assessing the logistics, excitement soon turned to **caution** and **concern**. How might we collaborate at a distance? Tossing up authentic practice versus health concerns, I felt torn. I chose the former, but I am still not comfortable with this choice (Melissa, October 2020).

What is this? So, it’s two months since I finished work after taking a voluntary redundancy when it was decided our campus will close. Our university was one of the first to run with redundancies and ‘voluntary **separations**' (starting in May), with others now snowballing. I’m at an age where I could ‘retire' – but wasn’t intending to for a few years, so still figuring out what is this life that I’m now living. It’s not a time to be looking for a university job in the arts and education in universities and I’m not in a position to **move** (Sue, October 2020).


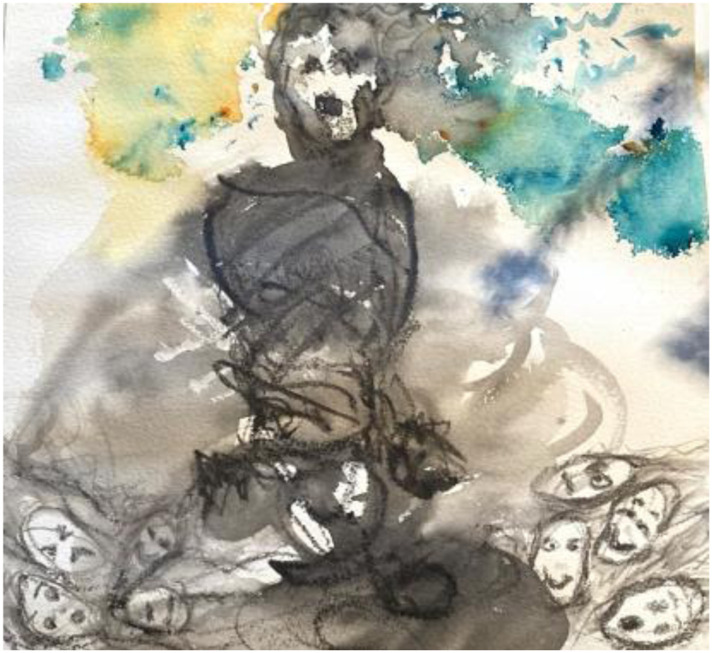


***The teacher ‘scream’ during COVID times Susan Davis,*** ***2020.***

Just as I am feeling **concern** regarding Sue’s situation, an item on a New Zealand news website this morning talks about all our universities introducing pay-**cuts** and job redundancies in response to the loss of funding since we can no longer rely on the income of international students. At the end of the piece, our Minister of Education states that the universities are not really in the dire financial situations that our VCs are portraying (Keogh, 2020). This made me think about Naomi Klein’s (2007) notion of ‘**shock** doctrine' whereby crises are utilised by corporates to capitalise on people’s disadvantage (Jenny, May 2020, Wellington).

Yesterday I was emailing a colleague who I haven’t seen for many months, reflecting on this strange year, tēnei tau tino rerekē. Someone in our zui (Zoom meeting) this morning mentioned that people are desperate to return to **‘normal'** yet it has become abundantly clear that we can never return to that pre-pandemic ‘normal'. I feel like we are on a very **scary**, **unknown** trajectory of exponentially multiplying crises of COVID, the exacerbating climate crisis, biodiversity extinction, and the last gasps of late neoliberal hyper-capitalism. Like Bruno Latour (2020), I am wondering where we will eventually land after this pandemic? (Jenny, 2nd October 2020, Wellington**).**

Yes, **scary** times! Jenny’s words in Wellington resonate with me in Wyoming. [Oct. 5, 2020] Today I’m most concerned about budget cuts at my university and within my college. Because of the devastating effects of COVID on our national and state economies, our university will need to make serious budget **cuts.** Our state legislature has told the Board of Trustees the extent of the cuts to our institution for the remaining fiscal year. In turn, the university president divvied up the needed cuts across each college on campus. How will these cuts impact colleagues? Colleagues may lose their livelihood (Cynthia).

We here in Aotearoa (NZ) are comparatively COVID-19 free at the moment. Our island and isolated status help protect our boundaries. We won’t, however, be free of the threat until the whole world is **free**. And now the so-called ‘leader of the free world' is sabotaging any hopes of dealing with the virus through his completely idiotic abandonment of science, compassion, and ethical responsibility. I obsessively watch the train-wreck of USA politics, I **grieve** for the over 200,000 lives that have been lost in the USA alone, and mourn for the onslaught to the world’s biodiversity that is incomprehensively irreparable (Jenny, October. 2020).

I have roles but no ‘position’, I have** identities** but limited positional power (Davies & Harre, 1990; Slocum-Bradley, 2009). The voice of the ‘former’ academic is one I am **cautious** of using. Beware of negativity and critique, lest one sound bitter and suffering from relevance deprivation. I’m still supervising research higher degree students, on a journal editorial board, marking theses, editing journal articles, and publishing a few, but … I apply for a creative business incubation program… I **agonise** over what to write… Who am I? Which roles, which identities, which stories, what face, what fronts, which acts, performance, and costumes? (Brock et al., 2021; Goffman, 1959) (Sue)


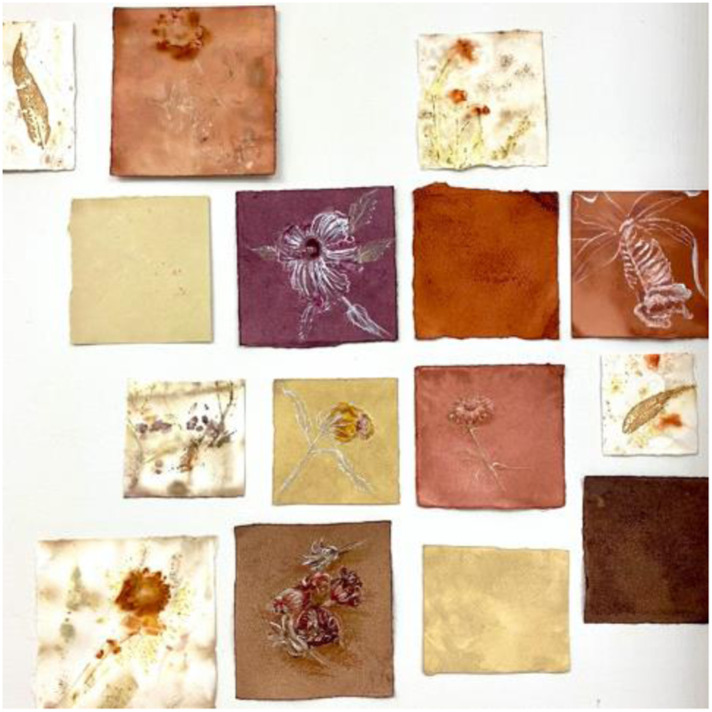


‘***Wagga wall’ paper installation, Susan Davis** **2021***

I have been wondering about how responding to the COVID-19 pandemic has changed us for better and for worse. For example, benefiting the **planet** through reducing emissions by moving to Zoom meetings and conferences, which also means that we lack the nuanced, sensorial, embodied connection that kanohi ki te kanohi (face-to-face) encounters provide. Our capacity to immediately **change** our most intimate daily routines and behaviours to protect our individual and collective well-being was impressively demonstrated. I wonder how we might harvest this same commitment to, and capacity for, immediate change in service of the **climate crisis**. But will we commit? (Jenny, October 25, 2020, Wellington).

In Aotearoa (New Zealand), we have just re-elected a Labour government, this time with a majority that enables them to govern alone, previously unheard of under our MMP electoral system. This provides a strong mandate for them to enact progressive policies to address the **climate crisis**, which our Prime Minister Jacinda Ardern has previously acknowledged as ‘our generation’s nuclear moment’. Whilst measures to alleviate the** damage** of the pandemic have been costly, I wonder if she will adhere to this **commitment** and enact the policies that require the urgent changes needed as she did during the immediate COVID-19 crisis? (Jenny, October 2020, Wellington).


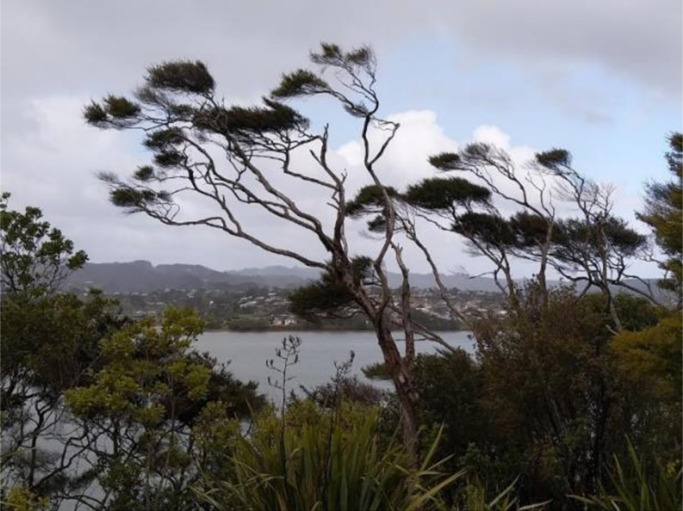


***View from Te Akau, looking across Whaingaroa harbour towards Raglan, Aotearoa. *** Photo: Jenny Ritchie

Te Akau is a tiny community on the opposite side of Whaingaroa harbour from the township of Raglan. I am here with my oldest son, his partner, and their one-month-old daughter. In this remote and very beautiful spot, the bush, trees, birds, and peaceful tidal harbour inlet belie the **craziness** of the year that is drawing to an end. Whilst COVID-19 rages across the rest of the planet, here in Aotearoa we continue to avoid a major outbreak. I wore a facemask for just the second time on my flight north. Our lives seem deceptively normal (Jenny, 26^th^ November, 2020).

Looking in the rear vision mirror, I see ‘the **unsettling**’. A movie crammed with unexpected and dispiriting happenings that jump around the screen and are barely connected. I reflect on the confusion felt when we first learnt that we wouldn’t be coming back to campus. Then the considerable struggle trying to embody the arts with my students whilst distanced. The essential non-verbal communication silenced by masks and overwhelmed by the smell of disinfectant. During this time, I learnt who I really am. Someone who relies on routine and who needs structure and predictability, but within parameters that allow for creativity (Melissa, May, 2022).

I felt ‘the **unsettling**’ viscerally. Too much sitting, disturbed sleep, and inner anger. But I have a place to go. High up in the mountains where the waterfalls and tall trees have stayed strong for centuries and are there to greet me without fail. Where the black cockatoos come flying to the creek like clockwork every dusk to drink collectively, before one gives the signal, and they soar spectacularly out over the valley and into the night. They provide **peace** and certainty. This ancient rainforest has seemed to withstand COVID, gun violence, potential world war, and climate change for now (Melissa, 31/05/2022).


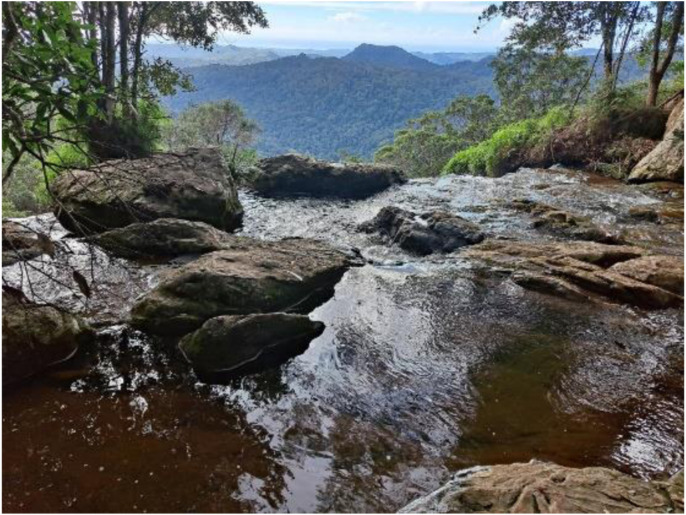


***Springbrook National Park in the Gold Coast Hinterland, Queensland, Australia. *** Photo: Melissa Cain

Yesterday some of the writers of this article reflected on Melissa’s entry above, about how reconnecting with forests and beaches has provided us with a sense of restoration during the past two years. The late Moana Jackson, a Māori legal scholar who has informed our understandings of pathways beyond colonisation, in one of his final contributions, *Decolonisation and the Stories in the Land* (2020), posited **restoration** as an alternative to decolonisation. This means the restoration of harmony in our relationships with Papatūānuku, Mother **Earth**, and between all peoples through conciliatory and consensual democracy. The forests can teach us about restoration (Jenny, June 18, 2022).


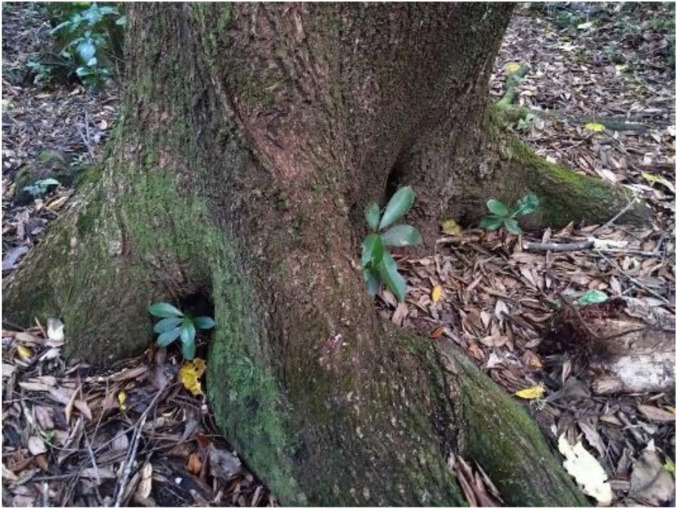


***New growth nurtured by old, Johnston Hill, Kārori, Wellington.*** Photo: Jenny Ritchie

We’re still working from **home** and in isolation here in Melbourne. Our Vice Chancellor decrees a week’s holiday to revive us a little. Spring has sprung, and the wattles are blooming. Only now can we leave our homes for two-hours respite per day; with mandated restrictions to stay within a five-kilometre radius, wearing masks and social distancing. This is the scenario for students and staff. Yet, the weather is uplifting, and I feel a sense of **hope** about being outside again. Universities are still teaching from home, but today, some Melbourne schools went back on campus after months of lockdown (Geraldine, 5/10/2020).

Seven months of lockdown and in amongst it my son has emergency surgery to remove his appendix – no visitors allowed. All went well as did a milestone birthday picnic for another son, celebrated in a park within our overlapping five-kilometre lockdown zones. Assignments role in for marking. Out of 120 students, 25 ask for extensions – most on account of COVID. As we round off the teaching year, **fatigue** is setting in; the long haul of lockdown is impacting us all. We are taking note, undertaking interactive Zoom sessions to tune in with each other with feedback and feed-forward initiatives (Geraldine, 12/10/2020).

COVID numbers are decreasing, only two cases announced in Melbourne today but 15 mystery cases. Will our restrictions lift soon? How will the University sector re-open again? Like other universities in Australia, we are now going through a process of ‘voluntary separation packages' (16/10/2020). We are out of stage 4 lockdown! We can travel within a 25k radius! Classes continue online until the end of this week. What a year – I’m pleased and **relieved** to have got through seven months of lockdown. A time to celebrate the **perseverance** and **creativity** of our students and co-teachers. What a huge effort! (Geraldine 6/11/2020).

Today we’re allowed to be ‘mask free' in open spaces, but masks must be worn in high-density situations. I’m feeling the air on my face, outside again – with **cautious optimism**. Eateries and pubs are opening up, but nursing – homes are still very restricted – so tough for seniors. We meet to plan teaching for semester 1 – how will we do it? Smaller cohorts? Social distancing? International teaching will still be online until late 2021. More changes afoot as we re-think room use, material sharing, and safety protocols; we are getting skilled at ‘change’ across modes of delivery and schedules (Geraldine, 23/11/2020).


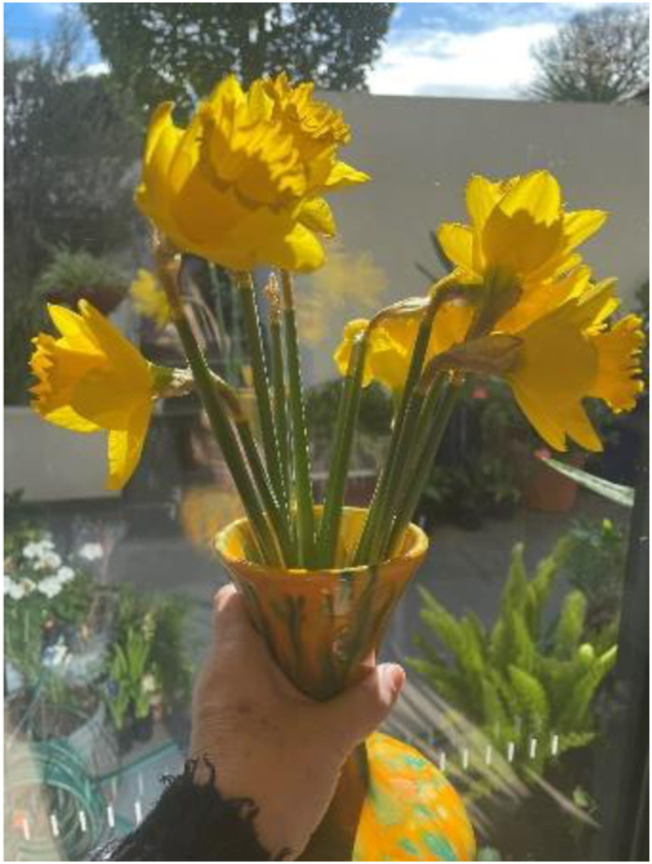


***Sending you a lockdown uplift today (email signature, Geraldine, 2020*** ***)***

**Looking back** on the long lockdown, I remember the gestures of outreach such as people adding images of flowers to email signatures to lift sagging spirits. Likewise, as educators we worked hard to build **pedagogies of kindness** (Clegg & Rowland, 2010) as a strategy to lift the learning benefits and well-being possibilities of students. Yet, how can inclusivity of kindness extend to inclusivity for the educator? Can the efforts behind this level of care be sustainable? How can a **politics of kindness** be reimagined to support educators by recognising and valorising the value of care work (Magnet et al., 2014)? (Geraldine, 15/9/2022).

This is a non-teaching week so I’m frantically designing new modules, catching up on research, and doing admin that has fallen by the wayside. **Moving** within the space while **listening** – differently; offers in-sight. In-sight foresight hindsight – feel as if they are knowledge, growing, and moving.

Age-old stories of knowledge that if you stop for long enough and pay attention, you will be able to see – differently. Stopping pausing **feeling** the dilemmas in the shadows

we can move.

here we are unstuck slippery. breathe inhale, exhale.

Pause. Notice. See. Hear. Feel. Know.

Angst, gratitude, sighs, kindness, cares.

(Kate, Melbourne 2/10/2020)

### A Look Back

I remember the days at work were long in the early stages of lockdown in Melbourne. I was really focused on my work, my students, research, and developing digital spaces for these to pivot into. In hindsight I should have been more focused on me and my **family**. But, as a geek and nerdy teacher, I have always loved apps and ed tech and I felt like my moment in the sun was here and I knew what to do. This was simultaneously a good thing and a bad thing as it affected me in tremendous ways (Kate).

It was fun to design, redesign, and shift teaching and student learning with agility and experience as a learning designer in new digital spaces.

The **harder** I worked, the longer the days got. The more I wound myself into what I was doing, the harder it was to get out of and see what I was doing and what I was **falling** into.

It **broke** me, as an overload of teaching and course coordination mixed with a love of digital placemaking, and not getting a role I applied for in digital leadership, all hit me like a tonne of bricks (Kate).

In 2021, I designed and redesigned as more pivots shifted us around and around. I went onto campus then home, into new digital spaces I had access to, piloting and playing within with students. I love **disruption**. I am keen to break things and put them back together and enjoyed this process of my work, but digital disruption is hard because it’s speculative work. The last two years have **changed** me. As a mother, partner, teacher, and person. As 2022 looks to be a new unknown I will disrupt and pivot, but with a harder shell than before (Kate).

The ‘who am I?’ game continues. I have the opportunity to apply for a job in the university sector again, a similar role to before, … but I don’t. I know that a deep core of my identity is bound up with learning and teaching. I am motivated by **creating opportunities** with and for others, whether students or artists. Am I still an academic? On my ‘Linked In’ profile, that is no longer what I lead with. I have become more **comfortable** with multiple roles and positionality, of **creating** my own quilt of storied experiences (Sue).


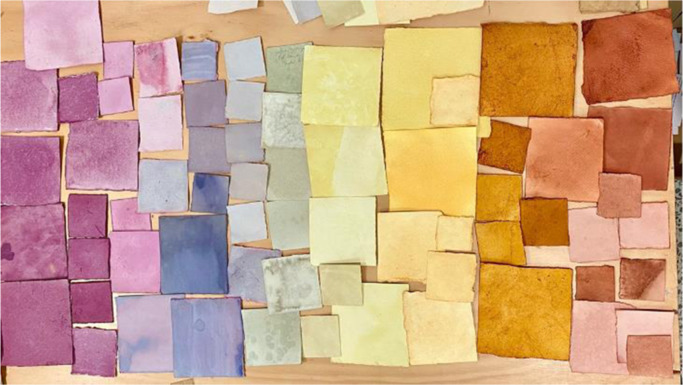


***Paper ‘quilt’ assemblage, Susan Davis*** ***2022***

## Conclusion

The pandemic added another burden for educators, on the top of ‘relentless pressure to perform’ where the standards for educational outcomes ‘are increasingly being set by the global education measurement industry’ (Biesta, 2019, p. 657). COVID-19 times have been the thorn which prompts us to consider ‘what counts *as* education and what counts *in* education’ (Biesta, 2019, p. 657). What we have discovered are the ‘I’ statements from both our participants and ourselves. The statements that reveal and illuminate ourselves as women and educators through impactful feminist intervention. As people committed to education in service of social and ecological well-being.

‘I am more focused on the emotional and sociocultural aspects of learning’.

‘I value certainty but can move forward in uncertain times’.

‘I can be flexible, but can’t cope with plans changing last minute’.

‘I can make the best of things and learn regardless of the obstacles’.

‘I have become a wild/flower woman’.

‘I really care about my students, and there are many ways to reach students’.

‘I am motivated by creating with others and by seeing others succeed’.

‘I should have been more focused on me and my family’.

‘I learnt who I really am’.

‘I am now okay’.

We can’t put these statements on our resumes or claim them as evidence in our performance reviews; but still we wonder. ‘What if our national and institutional priorities enabled us to consistently prioritise human **relationships** over scholarship, grant funding, and metrics? What if our job descriptions and promotion criterion meant that we would be able to count **connection, collegiality, creativity** and **compassion** as more valuable than our citations?’ (Cain & Fanshawe, 2021, p. 130). This **relationality** counts both *as* and *in* education. We now know that should uncertainty strike again, the security of the quilting antidote is there for us (Melissa).
